# Racial Impact on Inpatient Stroke Quality of Care in Two Community Hospitals

**DOI:** 10.3390/jcm12247654

**Published:** 2023-12-13

**Authors:** Haitham M. Hussein, Mai-Kau Yang, Solmaz Ramezani, Rishi Sharma, Omair ul haq Lodhi, Yaroslav Owens-Pochinka, Jinci Lu, Ahmed Elbokl

**Affiliations:** 1 Department of Neurology, University of Minnesota, MMC 295, 420 Delaware Street SE, Minneapolis, MN 55455, USAsramezan@umn.edu (S.R.); sharm415@umn.edu (R.S.); lodhi012@umn.edu (O.u.h.L.); pochi002@umn.edu (Y.O.-P.); lu000219@umn.edu (J.L.); 2 Institute of Health Informatics, University of Minnesota, Minneapolis, MN 55455, USA; elbokla@umn.edu; 3 Department of Neurology, Ain Shams University, Cairo 11517, Egypt

**Keywords:** ischemic stroke, quality of care, racial disparity

## Abstract

Introduction: This analysis was conducted as a part of a quality improvement project aiming at identifying racial disparity in inpatient stroke quality of care. Methods: The Get With The Guidelines (GWTG) database was used to identify all patients discharged with any stroke diagnosis between January and December 2021. An additional chart review was conducted to ensure the accuracy of racial/ethnic categorization. The sample was dichotomized into white vs. non-white groups and compared with univariate analysis. Results: The study sample comprised 1408 encounters (1347 patients) with Mean age of 71 ± 15 years, 51% women, 82% white patients, 15% non-white patients, 72% acute ischemic stroke (AIS); 15% transient ischemic attack (TIA), 9% intracerebral hemorrhage (ICH), 3% subarachnoid hemorrhage (SAH), and 1% stroke not otherwise specified. Non-white patients were younger and had fewer concomitant diagnoses, a lower proportion of TIA, and a higher proportion of ICH (*p* = 0.004). In the AIS cohort, compared to white patients, non-white patients had less frequent ambulance (*p* = 0.009), arrived at the hospital later than white patients (7.7 h longer; *p* < 0.001), had more severe strokes, and had less frequent IV thrombolysis utilization (7% vs. 13%; *p* = 0.042). Similarly, in the TIA cohort, non-white patients’ utilization of EMS was lower than that of white patients, and their hospital arrival was delayed. In the ICH cohort, non-white patients were younger and had a lower frequency of atrial fibrillation and a non-significant trend toward higher disease severity. The SAH cohort had only eight non-white patients, six of whom were transferred to a higher level of hospital care within a few hours of arrival. Importantly, the hospital-based quality metrics, such as door-to-CT time, door-to-needle time, and the Joint Commission stroke quality metrics, were similar between the two groups. Conclusions: There is a racial disparity in the pre-hospital phase of the stroke chain of survival of non-white patients, impacting IV thrombolysis utilization. The younger age and worse lipid profile and hemoglobin A1c of non-white patients suggest the need for better preventative care starting at a young age.

## 1. Introduction

Racial disparity in stroke outcomes has been well documented [1,2] and highlighted in an important statement by the American Heart Association (AHA) more than a decade ago [3]. While many of the factors contributing to this phenomenon, such as social determinants of health (SODH) [4,5], systemic racism [6], suboptimal risk factor control [7,8], and access to care [9], are not under the control of the stroke center where patients are admitted for their stroke, the acute phase of stroke care is. Racial disparity in acute stroke care has also been reported with lower rates of IV thrombolysis (IVT) [10,11] and mechanical thrombectomy (MT) utilization [12,13], longer door-to-needle (DTN) [14,15] and longer door-to-groin puncture (DTG) times [16], and less adherence to stroke quality metrics [17].

Our healthcare organization operates nine hospitals in the state of Minnesota with a stroke network that covers all nine hospitals and serves over 3500 stroke admissions annually. In 2022, three of our medical students wrote papers on racial disparity in stroke as part of their Problems in Neurology class. Using data from one of our hospitals, they identified a significant disparity in IVT utilization between white and African-American patients (11.1% vs. 5.1%, *p* < 0.001) [18]. While we had expected to find some difference between the two groups, the magnitude of the difference was surprising, and the data available did not allow for the exploration of possible causes. That analysis triggered a quality improvement project to better characterize and address the racial disparity in stroke care in our organization. Our current study was conducted as the first stage of this quality improvement project. 

## 2. Materials and Methods

Our stroke network is composed of one academic comprehensive stroke center (CSC) and 8 community hospitals, including one thrombectomy-capable stroke center (TSC), one primary stroke center (PSC), and 6 stroke-ready hospitals. All hospitals follow the same internal stroke care guidelines, which are derived from the AHA guidelines, and are served by the same academic vascular neurology team. We selected 2 of our community hospitals to extract the sample for this analysis. The first hospital is a TSC in Minneapolis, which provides stroke care for all patients except severe cases of SAH, who are typically transferred to the CSC. The second is a PSC in St. Paul, which provides care for all stroke patients except large vessel occlusion who require MT and who are transferred to either the CSC or TSC, ICH patients who require neurosurgical procedures, and all SAH who are transferred to CSC. These two hospitals were chosen because of a high stroke volume and based on geographical location to ensure the inclusion of a diverse population.

We used our institutional Get With The Guidelines (GWTG) stroke registry to identify the study sample and extract the relevant variables for analysis. GWTG is a nationwide database maintained by the AHA, which contains extensive information, including pre-hospital care, vascular risk factors, acute stroke treatment, hospitalization outcome, and various quality metrics. GWTG is used in daily operations for quality monitoring. Dedicated trained nurses, designated as data abstractors, update our institutional GWTG database with new patients on a daily basis using the information included in the medical records, including the race/ethnicity variables, which are based on patients’ self-identifications. GWTG data is subjected to regular quality reviews for accuracy internally by the stroke coordinators and externally by The Joint Commission, which grants our hospitals their stroke certifications. 

For this retrospective analysis, we selected adult patients discharged with a stroke diagnosis, including acute ischemic stroke (AIS), transient ischemic attack (TIA), intracerebral hemorrhage (ICH), and subarachnoid hemorrhage (SAH), in the calendar year 2021. Inclusion criteria were age 18 years or older and being admitted through the emergency department (ED). We excluded patients who had their stroke during their hospitalization (inpatient strokes), patients who were suspected of having a stroke on admission but ended up with a different diagnosis on discharge (stroke mimics), and patients with incomplete GWTG records. 

We used the GWTG database to collect and compare the following variables between white and non-white patients: age; sex; race/ethnicity; vascular risk factors; mode of arrival; initial vital signs; stroke severity scales (National Institutes of Health Stroke Scale [NIHSS] for AIS and TIA, and Glasgow Coma Scale [GCS] for ICH and SAH); ambulation status prior to index stroke, which reflects baseline functional state; AIS treatments (IVT and MT); acute care quality metrics (e.g., DTN and DTG times); laboratory data (lipid profile, hemoglobin A1c, random serum glucose); brain and vascular imaging performance; electrocardiogram and echocardiogram performance; length of stay; hospital mortality; ambulation on discharge; and discharge disposition. Further retrospective review of the electronic medical records (EMR) was needed to confirm the accuracy of the diagnosis and the race/ethnicity documented in GWTG and to abstract other variables related to race/ethnicity that are not part of GWTG: country of origin; primary language; and utilization of interpreters. 

This study’s aim was to assess whether there was a difference in stroke quality of care between white and non-white patients. Patients with undocumented race/ethnicity were categorized as non-white if their primary language was not English, their country of origin was not the US, or they needed an interpreter. The two study groups were compared using univariate analysis. Patients with unknown or undocumented race were included in the overall analysis but excluded from the comparative analysis. The following quality measures were compared between the two groups in all 4 stroke subtypes: last known well (LKW)-to-arrival time; door-to-CT performance time; mode of arrival (ambulance, private, transfer); documentation of stroke severity; length of stay; hospital mortality; discharge destination; ambulation on discharge; 30-day readmission; and 30-day mortality. Moreover, subtype-specific quality metrics were compared between the two study groups as follows:

AIS:
  - IVT utilization rate;
  - MT utilization rate;
  - DTN time;
  - DTG time;
  - The Joint Commission (TJC) stroke quality measures: STK1 (venous thromboembolism [VTE] prophylaxis); STK2 (discharge on antithrombotic therapy); STK3 (anticoagulation therapy for atrial fibrillation/flutter); STK4 (thrombolytic therapy); STK5 (antithrombotic therapy by end of hospital day 2); STK6 (discharged on statin medication); STK8 (stroke education); and STK10 (rehabilitation assessment);
TIA:
  - Completion of TIA workup: (1) brain magnetic resonance imaging (MRI); (2) vascular imaging of the head and neck with magnetic resonance angiography (MRA) or computed tomographic angiography (CTA); (3) echocardiogram transthoracic and/or transesophageal; (4) electrocardiogram; (5) lipid panel; (6) hemoglobin A1c. A scale from 0 to 6 was developed and calculated for each patient. A score of 6 out of 6 indicates performance in all 6 TIA tests;
ICH:
  - TJC quality measures: STK1, STK8, and STK10;
SAH:
  - Performance of vascular imaging (CTA, MRA);
  - TJC quality measures: STK1; STK8; and STK10.

The mean and standard deviation (SD) were reported for continuous variables and compared with the *t*-test after testing for normality of data distribution using the Q–Q plot and Shapiro–Wilk test. The median and interquartile range (IQR) were reported for continuous non-normally distributed variables and for non-parametric variables and were compared with the Mann–Whitney test. Frequency/percentages were reported for the categorical variables and were compared with the chi-squared test (or Fisher’s exact test if chi-squared assumptions are not met).

Missing data was identified and listed in Supplementary Materials (Tables S1–S5). Patients with missing data were excluded from the analyses of the variables they were missing but included in the analyses of the variables they had available. All *p*-values were 2-sided, with statistical significance at 0.05. Statistical analysis was performed using SciPy Fundamental Algorithms for Scientific Computing in Python. Plots and graphs were produced using Matplotlib and Seaborn data visualization libraries in Python.

This study’s concept and methodology were reviewed by the institutional review board, which deemed it to be a quality improvement project and allowed it to proceed without requiring full IRB review or subjects’ consent. 

## 3. Results 

**Figure 1:**
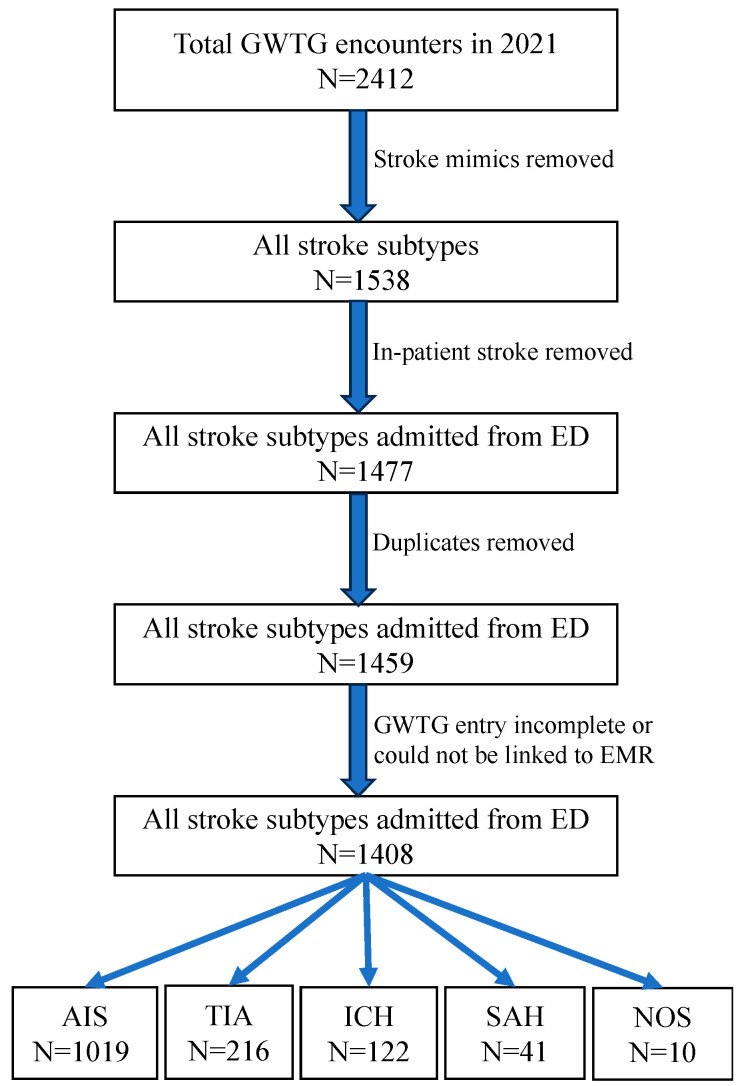
Study sample identification and categorization by stroke subtype.

There were 2412 encounters in the GWTG database within the study period. After excluding 874 stroke mimics, 61 inpatient strokes, 18 duplicate entries, and 51 records that could not be linked to EMR or were incomplete, the remaining 1408 encounters of 1347 patients represented the study sample (Figure 1).

**Figure 2:**
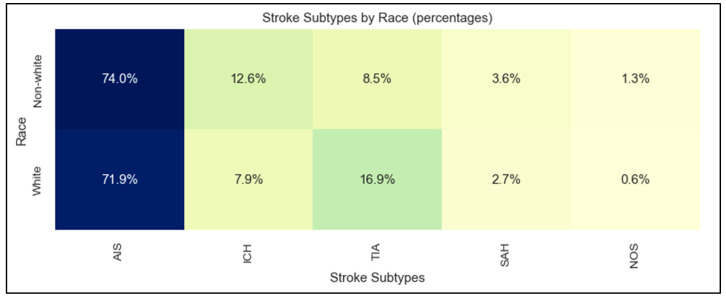
Stroke subtypes by race. AIS: acute ischemic stroke; TIA: transient ischemic attack; ICH: intracerebral hemorrhage; SAH: subarachnoid hemorrhage; NOS: not otherwise specified.

The study sample had a mean age of 71 ± 15 years, 51% women, none of whom were pregnant, 82% white patients, 10% Asian, 4% Black, 1% Hispanic, 11% using non-English primary language, and 6% requiring interpreters. Stroke subtypes were 72% AIS, 15% TIA, 9% ICH, 3% SAH, and 1% stroke not otherwise specified. Table 1 shows the basic demographic characteristics of the four stroke subtypes. The distribution of stroke subtypes was significantly different between white and non-white patients, with more hemorrhagic stroke (ICH and SAH) in non-white patients and more TIA in white patients (*p* = 0.004; Figure 2).

Regarding AIS cohort (1019 encounters; 977 patients), compared to white patients, non-white patients were significantly younger (median (IQR) 66 (57–73) vs. 75 (64–85) years; *p* < 0.001), had fewer documented concomitant diagnoses (four [two–five] vs. four [three–five]; *p* = 0.042), more patients with diabetes (45% vs. 27%; *p* < 0.001), higher hemoglobin A1c (6.3 (5.6–8.9)% vs. 5.7 (5.4–6.3)%; *p* < 0.001), especially in diabetics (8.5 (7–10)% vs. 7.1 (6.3–8.1)%; *p* < 0.001), fewer patients with dyslipidemia diagnosis (48% vs. 60%; *p* = 0.004) yet higher median LDL (99 (75–133) vs. 84 (61–116) mg/dL; *p* < 0.001), fewer atrial fibrillation (11% vs. 21%; *p* = 0.004), more history of prior stroke (35% vs. 25%; *p* = 0.006), lower frequency of emergency medical services (EMS) utilization (32% vs. 44%; *p* = 0.009), longer median time from LKW to arrival (856 (263–1921) vs. 393 (93–1188); *p* = 0.001), higher mean diastolic blood pressure (DBP; 87 (78–99) vs. 84 (74–96) mm Hg; *p* = 0.033), higher initial NIHSS (3 (1–6) vs. 2 (0–6); *p* = 0.023), and higher initial blood glucose (131 (109–188) vs. 115 (100–143) mg/dL; *p* < 0.001).

IVT utilization rate was lower in non-white patients (7% vs. 13%; *p* = 0.042); however, the rate was not statistically different when limited to patients presenting within 3.5 h from LKW (32% vs. 42%; *p* = 0.433). The reasons for not administering IVT were documented in 78% of white and 88% of non-white patients. The most common IVT exclusion reason for both groups was the inability to determine eligibility, usually due to unknown LKW time or inability to confirm inclusion/exclusion criteria (white 316 (51%); non-white 75 (67%)), while patient/family refusal was only documented in seven (1.1%) of white and two (1.8%) of non-white patients. See Table S6. 

In terms of hospitalization outcome, compared to white patients, non-white patients had fewer hospital mortality (1% vs. 5%; *p* = 0.029) and higher frequency of home discharge (69% vs. 58%; *p* = 0.011) despite having similar ambulatory status (*p* = 0.927). Other quality metrics, including arrival-to-CT time, DTN time, DTG, MT utilization rate, and all STK metrics, were not statistically significant (Table 2). Of note, there was no association between LKW time to arrival and DTN time (Pearson’s correlation coefficient −0.09; *p* = 0.33; Figure S1).

Similar to the AIS cohort, the non-white patients in the ICH cohort (122 encounters; 121 patients) were younger (57 (43–63) vs. 74 (64–82) years; *p* < 0.001), had fewer documented concomitant diagnoses (two [one–four] vs. four [three–five]; *p* = 0.003), fewer patients with dyslipidemia (29% vs. 58%; *p* < 0.011), and fewer patients with atrial fibrillation/flutter (0% vs. 18%; *p* = 0.012). Initial SBP, initial blood glucose, and intubation in the ED were numerically higher in the non-white group but were not statistically significant (Table 3). The in-hospital mortality rates, discharge disposition, and ambulatory status on discharge were not statistically different.

In the TIA cohort (216 encounters; 212 patients), compared to white patients, non-white patients were younger (77 (64–84) vs. 59 (51–77) years; *p* = 0.003), had lower frequency of EMS utilization (11% vs. 38%; *p* = 0.016), higher hemoglobin A1c (6.1 (5.9–6.2) vs. 5.5 (5.3–5.9); *p* = 0.002), and higher frequency of independent ambulation on discharge (93% vs. 87%; *p* = 0.011). No other characteristics were significantly different between the two groups, including TIA workup completion (Table 4).

Lastly, in the SAH cohort, which is the smallest cohort (41 encounters; 40 patients) with only eight non-white patients, six of whom were transferred to a higher level-of-care hospital, we could only make the following observations: compared to white patients, non-white patients had higher diastolic blood pressure (DBP; 81 (74–106) vs. 114 (91–133) mm Hg; *p* = 0.038), less frequently documented GCS (38% vs. 94%; *p* = 0.002), and a non-significant trend toward higher frequency of intubation in the ED (57% vs. 19%; *p* = 0.063). Other characteristics were not significantly different between the two groups, including the performance of vascular imaging (Table 5).

## 4. Discussion

In this retrospective analysis of patients admitted with stroke, we found a racial disparity in the pre-hospital phase of care (less EMS utilization and delayed arrival) and vascular risk factor control (dyslipidemia and diabetes mellitus). However, the quality of care delivered at the hospitals was largely similar between the white and non-white patients. 

Our study’s racial composition is unique in that out of the 15% non-white patients, 10% were Asian, and only 4% were black. The relatively high Asian population is due to the high concentration of the Hmong population in the catchment area of the study hospitals, especially in the city of St. Paul, where 19% of the population is Asian [19]. Stroke in Hmong Americans was first characterized in a recent retrospective study [20]. There are multiple similarities between this current analysis and stroke in Hmong Americans, which we will highlight in the relevant sections of this discussion. 

We observed that the median age of non-white patients was 11 years younger than that of the white patients, which is the same difference observed between Hmong Americans and white patients [20]. Non-white patients with ICH had the youngest median age compared to non-white patients of other stroke subtypes. We also observed that the proportion of ICH was higher in the non-white group, which is consistent with findings in Hmong Americans [20], other Asian ethnicities, such as Japanese [21] and Chinese [22], and Asian Americans in general [23,24]. This means that a larger proportion of younger non-white patients suffer from ICH, which is a disease with higher morbidity and mortality [25] and, unlike AIS, has no effective therapeutic intervention [26].

Our most important observation is the break in the stroke chain of survival at its first link. The stroke chain of survival starts with symptom recognition and calling 911, which activates EMS, leading to emergent transport of patients to the appropriate hospital and increasing the chances of providing reperfusion therapies [27]. Overall, the rate of EMS utilization in non-white patients was lowest in TIA, had the least severe stroke subtype, and was highest in the most severe stroke subtypes, ICH and SAH. These observations raise the concern that non-white patients have a lower tendency to seek emergent care in cases of mild or transient symptoms, which would be a missed opportunity in averting a disabling stroke. It is well known that the risk of stroke is high after TIA. A recent analysis of the Framingham study calculated an adjusted hazard ratio of 4.37 in TIA patients suffering a subsequent stroke compared to matched control participants who did not have a TIA [28]. This is why the AHA guidelines recommend rapid evaluation of TIA and initiation of secondary stroke prevention [29]. Early arrival is also important for AIS to increase the chances, maximize the benefits, and minimize the complications of IV thrombolysis administration [30].

The low rate of EMS utilization in non-white patients with AIS and TIA and the significant delay in arrival is similar to the observed delay in Hmong Americans [20] and racial minorities in general [31,32]. We recently conducted a stroke health literacy survey within the Hmong community and found that while 95% of respondents could name at least one correct stroke symptom, only 78% indicated that the first action they would take in the case of a stroke would be to call 911, while other respondents indicated that they would call an elder, call their doctor, drink water, take an aspirin, call 911 only if symptoms are severe, and other behaviors that are not helpful in the case of a stroke emergency [33]. We also learned from that survey that there is no equivalent word for ‘stroke’ in the Hmong language despite the familiarity of the respondents with stroke symptoms, and the anecdotal reports from Hmong community leaders indicate that many deaths in the Minnesota Hmong community were related to stroke [34]. Therefore, the language and health literacy gap could explain, at least partially, the difference in EMS utilization. To address this health literacy gap, we recently published stroke educational material cross-culturally adapted to Hmong [35]. Another important factor is ambulance cost. A recent analysis showed the rising cost of ambulance utilization from 2007 to 2018 among Medicare beneficiaries (up to USD 1700 per ride) [36], which may deter individuals of lower socioeconomic status from seeking EMS care. We also heard from the Hmong community during various community engagement events that some Hmong individuals did not feel comfortable interacting with any person in a uniform owing to prior historical trauma. 

EMS utilization rates were not different between white and non-white patients with ICH and were higher for both groups compared to their AIS counterparts. This might be related to the higher disease severity, given the non-significantly higher proportion of intubation in the ICH group compared to the AIS group. Similarly, the time from LKW to ED arrival was not statistically different between white and non-white ICH patients, although the median was numerically higher in the non-white group (about 4 h longer). These findings resemble those of stroke in Hmong Americans except that the EMS utilization was actually significantly higher, and the disease severity, as measured by NIHSS and GCS, was significantly higher in the Hmong-American patients compared to white patients [20].

Numerous studies have reported the racial disparity in IVT utilization rate [1,10,11,37,38]. We observed the same phenomenon (7% in non-white vs. 13% in white; *p* = 0.042), which was not surprising given the delay in ED arrival in the case of the non-white group. This finding is also similar to that of the stroke in Hmong Americans study in which the utilization rate was 6% in Hmong patients and 14% in white patients (*p* = 0.03496) [20], and a nationwide GWTG analysis that included 64,337 Asian American patients and showed lower IVT utilization rates compared to white patients (odds ratio 0.95; 95% confidence interval 0.91–0.98; *p* = 0.003) [38]. When we limited the comparison to patients who presented within 3.5 h from LKW, allowing the clinical team one hour for evaluation and treatment, there was no statistical difference between white and non-white groups, which further supports the relationship between delay in arrival and the low rate of IVT. 

It is important to point out that the two most common reasons for not administering IVT were similar in both groups. The first is the inability to determine eligibility, which includes patients with unclear diagnoses, patients with unknown time of onset, and patients in whom the inclusion/exclusion criteria cannot be confirmed. The second is mild stroke deficit. These two reasons were documented in over three-quarters of the patients who were not treated with thrombolysis in each group. The rate of refusal of IVT was similarly low in both groups, unlike other studies showing a higher rate of refusal in racial minorities [39,40]. However, in those studies, the finding was mainly driven by African-American patients, who represented only 4% of our AIS cohort. 

Aside from the lower IVT utilization rate attributable to delay in arrival, the hospital-based quality metrics were not different between white and non-white patients, with the exception of the GCS documentation in SAH, which was lower in non-white, probably related to higher disease severity, intubation, and transfer to CSC (median length of stay was 3 h). We did not find a difference in the MT utilization rate. While this may be related to the small sample size, the extended treatment window of MT (up to 24 h from LKW) [41] may have allowed for the intervention in non-white patients who arrived beyond the IVT window. Other studies have shown lower rates of MT in racial minorities, but these were primarily driven by African-American and Hispanic patients [11,12,13]. TIA workup was similar between white and non-white groups. A recent study showed suboptimal care of African-American TIA patients compared to white patients with lower rates of brain and vascular imaging except in those who have been cared for by a neurologist [42].

We observe important differences in vascular risk factor control between white and non-white groups in the AIS group. Hemoglobin A1c and lipid profile were worse in the non-white group. The same pattern can be observed when the analysis is limited to those with known diabetes mellitus in the case of hemoglobin A1c and dyslipidemia in the case of lipid profile. These observations are consistent with many studies indicating the undertreatment of cardiovascular risk factors in racial minorities. For example, an analysis of the 2013 Medical Expenditure Survey showed lower diabetes quality of care in racial minorities, particularly hemoglobin A1c monitoring, which was attributed to socioeconomic state and access to healthcare [43]. Another study found lower rates of utilization of new antidiabetic drugs irrespective of socioeconomic status [44]. As for dyslipidemia, data from the Behavioral Risk Factor Surveillance System showed that lower socioeconomic status, lack of healthcare access, and language barriers explained most of the racial and ethnic disparities in cholesterol screening [45]. In a recent study that analyzed National Health and Nutrition Examination Survey (NHANES) data from 2007 to 2018, an improvement in lipid profile was noted in all racial/ethnic groups except the Asian group [46]. In fact, SDOH entirely accounted for the disparity in cardiovascular mortality in another NHANES analysis using data from 1999 to 2018 [47].

We do not have data comparing hypertension control between our two study groups prior to the index stroke. However, racial disparity in hypertension is well documented [48,49]. Relevant to our study is a recent analysis of 259,824 hypertensive ambulatory patients in Minnesota from 2015 to 2019; the rate of uncontrolled hypertension was highest in Hmong, African-American, and Somali patients [50]. 

Furthermore, the COVID-19 pandemic could have contributed to the disparity in vascular risk factor control observed in our sample. Management of vascular risk factors was noted to be suboptimal during the pandemic, especially in African-American patients [51]. A recent publication highlighted the nationwide worsening hypertension control post-COVID-19 and that certain racial minorities, such as African Americans and Asian and Hispanic minorities, were more severely impacted [52]. Another study showed a higher incidence of diabetic ketoacidosis in African-American diabetic patients [53]. While the effect of COVID-19 was not examined in our study, which is limited to the calendar year 2021, one may speculate that the racial disparity in hypertension and other vascular risk factor control has increased because of the pandemic contributing to the racial gap we observe in the risk factor control in our sample. 

Our study has important limitations, including the retrospective design and the small sample size. The data source is a quality database, not a research one. We did not have control over the GWTG data abstraction and only accessed the charts to confirm the race and other relevant variables but did not verify the accuracy of other variables or complete any missing values. Some patients were missing the race/ethnicity categorization in the GWTG and the medical records and were assumed to be non-white if their country of origin was not the United States or if their primary language was not English. We did not have data on important SODH that have been found to be important predictors of stroke incidence and outcome, such as income, level of education, employment, housing, and insurance status [1,4]. We were unable to follow the recommended disaggregation of races/ethnicities [54] and combined all non-white patients in one group because of the small sample size, which might have obscured important inter-racial differences. 

This analysis is part of quality improvement efforts to address racial disparity in our stroke system of care. Our plan to mitigate the disparity has two main components. The first is to address the break in the stroke chain of survival by engaging with the racial minority communities to improve stroke health literacy and encourage the use of EMS, and the second is to engage with local healthcare systems and primary care providers to improve primary prevention. We have already shared the results of this study internally through multiple lectures and conferences on the topic, including one lecture in which one of our stroke survivors from a racial minority shared the stroke admission experience and provided feedback about areas of improvement. We also shared the results of this analysis with the Cardiovascular Health Alliance of the Minnesota Department of Health. We participated in a health-focused podcast with one of our stroke survivors from a racial minority and discussed the issue of racial disparity in stroke. We also have upcoming speaking events targeting primary care providers in clinics that provide care to racial minority groups. At the community level, we have reached out to multiple social service organizations of racial minority communities. We have an ongoing research project utilizing the University of Minnesota Mobile Health Initiative to identify individuals with elevated blood pressure and provide them with self-monitoring blood pressure measuring devices. After the results of this analysis, we decided to add stroke education to the educational material we are providing. 

## 5. Conclusions

The disparity in acute stroke care is mainly driven by delayed hospital arrival and low EMS utilization. Efforts are needed to engage with racial minority communities to improve health literacy and encourage EMS utilization. Some vascular risk factor control is worse in non-white patients, suggesting the need for more rigorous primary care. 

## Figures and Tables

**Table 1:** Basic demographic characteristics of the 4 disease states included in the analysis.

	AIS	TIA	ICH	SAH
Sample size	1019	216	122	41
Mean age	72 ± 15	72 ± 15	68 ± 17	67 ± 16
Female sex	51%	57%	44%	70%
Non-White	15%	7%	22%	20%
Countries of origin	United States 87% Laos 6% Russia 1% Myanmar 1% Vietnam 1% Cambodia 1%	United States 92% Laos 2% United Kingdom 1% Cambodia 1%	United States 82% Laos 4% Somalia 3% Cambodia 2% Philippines 2% Myanmar 2% Mexico 2%	United States 85%, Vietnam 10% Cambodia 3% Laos 3%
Non-English primary language	11%	5%	13%	15%
Need for interpretation	7%	2%	7%	5%

AIS: acute ischemic stroke; TIA: transient ischemic attack; ICH: intracerebral hemorrhage; SAH: subarachnoid hemorrhage.

**Table 2:** Univariate analysis comparing white and non-white groups of the acute ischemic stroke cohort.

	White	Non-White	*p*-Value
Sample size	860	173	
Age in years, median (IQR)	75 (64–85)	66 (57–73)	<0.001
Women, frequency (%)	424 (51%)	81 (48%)	0.713
Number of concomitant medical diagnoses, median (IQR)	4 (3–5)	4 (2–5)	0.042
Hypertension, frequency (%)	600 (72%)	119 (72%)	1.000
Diabetes mellitus, frequency (%)	224 (27%)	78 (45%)	<0.001
Dyslipidemia, frequency (%)	502 (60%)	79 (48%)	0.004
Atrial fibrillation/flutter, frequency (%)	175 (21%)	18 (11%)	0.004
Coronary artery disease, frequency (%)	186 (22%)	17 (10%)	0.001
Heart failure, frequency (%)	84 (10%)	11 (7%)	0.220
Current smoker, frequency (%)	111 (13%)	19 (12%)	0.610
Alcohol or illicit drug abuse, frequency (%)	66 (8%)	9 (5%)	0.347
Sleep apnea, frequency (%)	96 (12%)	11 (7%)	0.087
Overweight/obesity, frequency (%)	451 (54%)	84 (51%)	0.490
Prior stroke, frequency (%)	211 (25%)	58 (35%)	0.006
Family history of stroke, frequency (%)	87 (10%)	13 (8%)	0.387
Hemoglobin A1c (%), median (IQR)	5.7 (5.4–6.3)	6.3 (5.6–8.9)	<0.001
Hemoglobin A1c (%) in patients with known diabetes mellitus diagnosis, median (IQR)	7.1 (6.3–8.1)	8.5 (7.0–10.0)	<0.001
LDL (mg/dL), median (IQR)	84 (61–116)	99 (75–133)	<0.001
LDL (mg/dL) in patients with known dyslipidemia, median (IQR)	78 (55–111)	99 (75–134)	<0.001
HDL (mg/dL), median (IQR)	47 (38–58)	44 (37–54)	0.066
HDL (mg/dL) in patients with known dyslipidemia, median (IQR)	45 (38–58)	45 (40–54)	0.932
Triglycerides (mg/dL), median (IQR)	112 (81–161)	125 (85–184)	0.033
Triglycerides (mg/dL) in patients with known dyslipidemia, median (IQR)	114 (82–166)	132 (91–186)	0.047
Total cholesterol (mg/dL), median (IQR)	160 (132–194)	176 (144–210)	<0.001
Total cholesterol (mg/dL) in patients with known dyslipidemia, median (IQR)	154 (126–188)	180 (150–211)	<0.001
Ambulatory status prior to index stroke, frequency (%)			0.841
Independent	722 (91%)	146 (92%)	
With assistance	47 (6%)	9 (6%)	
Unable	27 (3%)	4 (3%)	
Mode of arrival, frequency (%)			0.009
EMS	342 (44%)	50 (32%)	
Private	279 (39%)	79 (51%)	
Hospital transfer	131 (17%)	26 (17%)	
Time from last known well to ED arrival in minutes, median (IQR)	393 (93–1188)	856 (263–1921)	0.001
Time from ED arrival to CT performance in minutes, median (IQR)	20 (15–28)	17 (14–20)	0.163
Door-to-needle time in minutes, median (IQR)	65 (46–88)	56 (41–132)	0.832
Door-to-groin time in minutes, median (IQR)	112 (101–123)	134 (101–190)	0.342
SBP in mm Hg, median (IQR)	155 (137–173)	158 (137–182)	0.114
DBP in mm Hg, median (IQR)	84 (74–96)	87 (78–99)	0.033
Blood glucose in ED in mg/dL, median (IQR)	115 (100–143)	131 (109–188)	<0.001
Initial NIHSS documentation, frequency (%)	625 (75%)	119 (72%)	0.477
Initial NIHSS score, median (IRQ)	2 (0–6)	3 (1–6)	0.023
Initial NIHSS score for IVT patients, median (IRQ)	6 (3–13)	7.5 (5.5–14)	0.546
24-h NIHSS score, median (IRQ)	1 (0–4)	3 (1–6)	<0.001
24-h NIHSS score for IVT patients, median (IRQ)	3 (1–8)	2 (1–6)	0.804
IV antihypertensive use in the ED, frequency (%)	76 (10%)	18 (12%)	0.554
Intubation in the ED	21 (3%)	1 (1%)	0.235
IV thrombolysis utilization rate, frequency (%)	111 (13%)	12 (7%)	0.042
IV thrombolysis utilization rate in patients presenting within 3.5 h, frequency (%)	102 (42%)	8 (32%)	0.433
IV thrombolysis administered at a different facility before transfer, frequency (%)	25 (5%)	3 (4%)	0.784
Mechanical thrombectomy utilization rate, frequency (%)	42 (5%)	5 (3%)	0.36
Length of stay in hours, median (IQR)	66.3 (41.7–112.6)	64 (45.1–113)	0.716
In-hospital mortality, frequency (%)	38 (5%)	1 (1%)	0.029
Discharge destination, frequency (%)			0.071
Home	459 (58%)	113 (69%)	
Rehab/nursing home	242 (30%)	35 (21%)	
Acute care hospital	55 (7%)	12 (7%)	
Hospice	28 (4%)	4 (2%)	
Against medical advice	10 (1%)	0	
Ambulatory status at discharge, frequency (%)			0.97
Independent	440 (61%)	93 (62%)	
With assistance	250 (35%)	51 (34%)	
Unable	31 (4%)	6 (4%)	
STK1: VTE prophylaxis	95%	94%	0.727
STK2: discharge on antithrombotic therapy	100%	100%	1
STK3: anticoagulation therapy for atrial fibrillation/flutter	99%	100%	1
STK4: thrombolytic therapy	92%	100%	1
STK5: antithrombotic therapy by end of hospital day 2	99%	98%	0.348
STK6: discharged on statin medication	99%	100%	1
STK8: stroke education	89%	91%	0.797
STK10: rehabilitation assessment	100%	100%	1

IQR: interquartile range; HDL: high-density lipoprotein; LDL: low-density lipoprotein; EMS: emergency medical services; ED: emergency department; CT: computed tomography; SBP: systolic blood pressure; DBP: diastolic blood pressure; NIHSS: National Institutes of Health Stroke Scale; IV: intravenous; VTE: venous thromboembolism; STK: The Joint Commission (TJC) stroke quality metrics (note that STK7 and STK9 have been retired by TJC).

**Table 3:** Univariate analysis comparing white and non-white groups of the intracerebral hemorrhage cohort.

	White	Non-White	*p*-Value
Sample size	91	27	
Age in years, median (IQR)	74 (64–82)	57 (43–63)	<0.001
Women, frequency (%)	41 (45%)	12 (43%)	1
Number of concomitant medical diagnoses, median (IQR)	4 (3–5)	2 (1–4)	0.003
Hypertension, frequency (%)	70 (77%)	17 (61%)	0.148
Diabetes mellitus, frequency (%)	20 (22%)	6 (21%)	1
Dyslipidemia, frequency (%)	53 (58%)	8 (29%)	0.011
Atrial fibrillation/flutter, frequency (%)	16 (18%)	0	0.012
Coronary artery disease, frequency (%)	18 (20%)	2 (7%)	0.154
Heart failure, frequency (%)	6 (7%)	1 (4%)	1
Current smoker, frequency (%)	6 (7%)	2 (7%)	1
Alcohol or illicit drug abuse, frequency (%)	11 (12%)	3 (11%)	1
Sleep apnea, frequency (%)	6 (7%)	1 (4%)	1
Overweight/obesity, frequency (%)	42 (46%)	17 (61%)	0.258
Prior stroke, frequency (%)	19 (21%)	7 (25%)	0.842
Family history of stroke, frequency (%)	17 (19%)	1 (4%)	0.069
Hemoglobin A1c (%), median (IQR)	7.3 (6.1–9.9)	8.2 (6.8–9.0)	1
Ambulatory status prior to index stroke, frequency (%)			0.531
Independent	75 (95%)	24 (100%)	
With assistance	2 (3%)	0	
Unable	2 (3%)	0	
Mode of arrival to the ED, frequency (%)			0.444
EMS	51 (57%)	13 (50%)	
Private	17 (19%)	8 (31%)	
Hospital transfer	21 (24%)	5 (19%)	
Time from last known well to ED arrival in minutes, median (IQR)	281 (83.5–838)	570 (72.5–792)	0.942
Time from ED arrival to CT performance in minutes, median (IQR)	27 (19–69.5)	41 (20–98.5)	0.3
SBP in mm Hg, median (IQR)	162 (138–190)	172 (140–202)	0.303
DBP in mm Hg, median (IQR)	90 (78–106)	85 (68–112)	0.412
Blood glucose in ED in mg/dL, median (IQR)	125 (106–162)	134 (128–174)	0.052
GCS score obtained on admission, frequency (%)	78 (86%)	21 (75%)	0.246
GCS score on admission, median (IQR)	15 (11–15)	12 (7–15)	0.308
IV antihypertensive use in the ED, frequency (%)	55 (65%)	19 (68%)	0.94
Intubation in the ED	10 (12%)	6 (22%)	0.222
External ventricular drain placement, frequency (%)	5 (6%)	4 (14%)	0.224
Decompressive craniectomy, frequency (%)	1 (1%)	1 (4%)	0.439
Hematoma evacuation, frequency (%)	5 (6%)	4 (14%)	0.224
Length of stay in hours, median (IQR)	84.4 (6.9–143.2)	41.3 (5.7–73.9)	0.069
In-hospital mortality, frequency (%)	17 (19%)	5 (18%)	1
Discharge destination, frequency (%)			0.830
Home	15 (20%)	7 (30%)	
Rehab/nursing home	29 (39%)	7 (30%)	
Acute care hospital	23 (31%)	7 (30%)	
Hospice	7 (10%)	2 (9%)	
Against medical advice	0	0	
Ambulatory status at discharge, frequency (%)			0.834
Independent	21 (42%)	5 (33%)	
With assistance	26 (52%)	9 (60%)	
Unable	3 (6%)	1 (7%)	
STK1: VTE prophylaxis	100%	92%	0.25
STK8: stroke education	91%	80%	1
STK10: rehabilitation assessment	100%	100%	1

IQR: interquartile range; EMS: emergency medical services; ED: emergency department; CT: computed tomography; SBP: systolic blood pressure; DBP: diastolic blood pressure; GCS: Glasgow Coma Scale; IV: intravenous; STK: The Joint Commission (TJC) stroke quality metrics (note that we only selected the STKs relevant to ICH); VTE: venous thromboembolism.

**Table 4:** Univariate analysis comparing white and non-white groups of the transient ischemic attack cohort.

	White	Non-White	*p*-Value
Sample size	212	19	
Age in years, median (IQR)	77 (64–84)	59 (51–77)	0.003
Women, frequency (%)	117 (55%)	11 (58%)	1
Number of concomitant medical diagnoses, median (IQR)	3 (2–5)	3 (3–4)	0.579
Hypertension, frequency (%)	128 (65%)	13 (68%)	0.984
Diabetes mellitus, frequency (%)	40 (20%)	6 (32%)	0.252
Dyslipidemia, frequency (%)	120 (61%)	9 (47%)	0.351
Atrial fibrillation/flutter, frequency (%)	35 (18%)	1 (5%)	0.210
Coronary artery disease, frequency (%)	42 (21%)	2 (11%)	0.376
Heart failure, frequency (%)	16 (8%)	0	0.371
Current smoker, frequency (%)	9 (5%)	2 (11%)	0.252
Alcohol or illicit drug abuse, frequency (%)	6 (3%)	1 (5%)	0.482
Sleep apnea, frequency (%)	23 (12%)	1 (5%)	0.703
Overweight/obesity, frequency (%)	107 (55%)	9 (47%)	0.717
Prior stroke, frequency (%)	31 (16%)	3 (16%)	1.000
Family history of stroke, frequency (%)	21 (11%)	0	0.228
Hemoglobin A1c (%), median (IQR)	5.5 (5.3–5.9)	6.1 (5.9–6.2)	0.002
Hemoglobin A1c (%) in patients with known diabetes mellitus diagnosis, median (IQR)	7.0 (5.8–7.8)	6.6 (6.1–7.5)	0.918
LDL (mg/dL), median (IQR)	87 (64–115)	103 (77–126)	0.357
LDL (mg/dL) in patients with known dyslipidemia, median (IQR)	79 (58–113)	113 (68–141)	0.300
HDL (mg/dL), median (IQR)	49 (42–62)	42 (35–68)	0.175
HDL (mg/dL) in patients with known dyslipidemia, median (IQR)	49 (44–59)	43 (39–64)	0.491
Triglycerides (mg/dL), median (IQR)	117 (84–164)	138 (90–180)	0.539
Triglycerides (mg/dL) in patients with known dyslipidemia, median (IQR)	119 (89–163)	127 (98–158)	0.942
Total cholesterol (mg/dL), median (IQR) + A34:A43	164 (139–201)	180 (162–211)	0.272
Total cholesterol (mg/dL) in patients with known dyslipidemia, median (IQR)	158 (135–200)	178 (151–212)	0.348
Ambulatory status prior to index stroke, frequency (%)			0.108
Independent	188 (99%)	17 (94%)	
With assistance	1 (1%)	0	
Unable	1 (1%)	1 (6%)	
Mode of arrival, frequency (%)			0.016
EMS	73 (38%)	2 (11%)	
Private	105 (54%)	16 (89%)	
Hospital transfer	16 (8%)	0	
Time from last known well to ED arrival in minutes, median (IQR)	132 (64–560)	165 (105–368)	0.203
Time from ED arrival to CT performance in minutes, median (IQR)	31 (17–64)	32 (29–39)	0.828
SBP in mm Hg, median (IQR)	156 (138–179)	152 (139–176)	0.587
DBP in mm Hg, median (IQR)	83 (75–94)	82 (70–100)	0.914
Blood glucose in ED in mg/dL, median (IQR)	108 (96–133)	117 (102–137)	0.421
Initial NIHSS documentation, frequency (%)	137 (70%)	13 (68%)	1.0
Initial NIHSS score, median (IRQ)	0 (0–1)	0 (0–1)	0.586
IV antihypertensive use in the ED, frequency (%)	8 (4%)	0	1.000
Intubation in the ED	0	0	1.000
TIA workup completeness score, median (IQR)	5 (3–6)	5 (2–6)	1.0
TIA workup			0.93
Complete	67 (34%)	7 (37%)	
Partial	128 (65%)	12 (63%)	
Not performed	1 (1%)	0	
Length of stay in hours, median (IQR)	22.8 (5.3–46)	26.75 (23.6–46.1)	0.548
In-hospital mortality, frequency (%)	0	0	1
Discharge destination, frequency (%)			0.484
Home	178 (91%)	18 (95%)	
Rehab/nursing home	12 (6%)	0	
Acute care hospital	6 (3%)	1 (5%)	
Hospice	0	0	
Against medical advice	0	0	
Ambulatory status at discharge, frequency (%)			0.011
Independent	86 (87%)	13 (93%)	
With assistance	13 (13%)	0	
Unable	0	1 (7%)	

IQR: interquartile range; HDL: high-density lipoprotein; LDL: low-density lipoprotein; EMS: emergency medical services; ED: emergency department; CT: computed tomography; SBP: systolic blood pressure; DBP: diastolic blood pressure; NIHSS: National Institutes of Health Stroke Scale; IV: intravenous; TIA: transient ischemic attack.

**Table 5:** Univariate analysis comparing white and non-white groups of the subarachnoid hemorrhage cohort.

	White	Non-White	*p*-Value
Sample size	31	8	
Age in years, median (IQR)	70 (57–84)	60 (48–75)	0.244
Women, frequency (%)	21 (68%)	6 (75%)	1
Number of concomitant medical diagnoses, median (IQR)	2 (1–3)	2 (1–3)	0.901
Hypertension, frequency (%)	14 (45%)	4 (50%)	1
Diabetes mellitus, frequency (%)	1 (3%)	2 (25%)	0.101
Dyslipidemia, frequency (%)	9 (29%)	4 (50%)	0.402
Atrial fibrillation/flutter, frequency (%)	3 (10%)	1 (13%)	1
Coronary artery disease, frequency (%)	3 (10%)	0	1
Heart failure, frequency (%)	1 (3%)	0	1
Current smoker, frequency (%)	4 (13%)	1 (13%)	1
Alcohol or illicit drug abuse, frequency (%)	1 (3%)	0	1
Sleep apnea, frequency (%)	3 (10%)	1 (13%)	1
Overweight/obesity, frequency (%)	13 (42%)	3 (38%)	1
Prior stroke, frequency (%)	4 (13%)	0	0.563
Family history of stroke, frequency (%)	3 (10%)	1 (13%)	1
Hemoglobin A1c (%), median (IQR)	5.6 (5.3–6.05)	5.4 (5.3–6.92)	0.98
Ambulatory status prior to index stroke, frequency (%)			1
Independent	28 (97%)	7 (100%)	
With assistance	1 (3%)	0	
Unable	0	0	
Mode of arrival to the ED, frequency (%)			0.184
EMS	15 (52%)	7 (88%)	
Private	12 (41%)	1 (13%)	
Hospital transfer	2 (7%)	0	
Time from last known well to hospital arrival in minutes, median (IQR)	808 (166–1749.5)	72 (59–1769.5)	0.862
Time from hospital arrival to CT performance in minutes, median (IQR)	64 (35.25–98.25)	33 (28–47.5)	0.112
SBP in mm Hg, median (IQR)	154 (131–168)	118 (103–192)	0.379
DBP in mm Hg, median (IQR)	81 (74–106)	114 (91–133)	0.038
Blood glucose in ED in mg/dL, median (IQR)	115 (94–157)	139 (123–150)	0.325
GCS on admission, median (IQR)	15 (12–15)	15 (9–15)	0.813
GCS documented on admission, frequency (%)	29 (94%)	3 (38%)	0.002
IV antihypertensive use in the ED, frequency (%)	8 (4%)	0	1
Intubation in the ED	6 (19%)	4 (57%)	0.063
External ventricular drain placement, frequency (%)	8 (26%)	2 (29%)	1
Performance of CTA or MRA	25 (81%)	6 (75.0%)	0.658
Aneurysm coiling, frequency (%)	7 (23%)	2 (29%)	1
Aneurysm clipping, frequency (%)	3 (10%)	0	1
Length of stay in hours, median (IQR)	27.6 (5.7–71.1)	3 (1.9–5.4)	0.035
In-hospital mortality, frequency (%)	2 (6%)	1 (13%)	0.508
Discharge destination, frequency (%)			0.481
Home	8 (28%)	1 (14%)	
Rehab/nursing home	4 (14%)	0	
Acute care hospital	16 (55%)	6 (86%)	
Hospice	9 (31%)	0	
Against medical advice	0	0	

IQR: interquartile range; EMS: emergency medical services; ED: emergency department; CT: computed tomography; SBP: systolic blood pressure; DBP: diastolic blood pressure; GCS: Glasgow Coma Scale; IV: intravenous; CTA: computed tomographic angiogram; MRA: magnetic resonance angiogram.

## Data Availability

Raw data are available from the corresponding author on reasonable request, following approval from the relevant data custodians.

## Supplementary Materials

The following supporting information can be downloaded at https://www.mdpi.com/article/10.3390/jcm12247654/s1, Table S1: Frequency and percent of missing data for the whole sample cohort; Table S2: Frequency and percent of missing data for the acute ischemic stroke cohort; Table S3: Frequency and percent of missing data for the transient ischemic attack cohort; Table S4: Frequency and percent of missing data for the intracerebral hemorrhage cohort; Table S5: Frequency and percent of missing data for the subarachnoid hemorrhage cohort; Table S6: Reasons for not administering IV thrombolysis; Figure S1: Scatterplot showing the last-known-well-to-arrival time and the door-to-needle time in patients who received IV thrombolysis.
