# Supplementary material for: Racial Impact on Inpatient Stroke Quality of Care in Two Community Hospitals
Source: J Clin Med. 2023 Dec 13;12(24):7654. doi: 10.3390/jcm12247654 (PMC10743521; doi:10.3390/jcm12247654)
Supplement: Supplementary file 1 [file jcm-12-07654-s001.zip › Supplemental Table S1-S6.pdf]

Supplementary Tables

Table S1: Frequency and percent of missing data for the whole sample cohort

Variable	Whole Missing	Whole Missing %	White Missing	White Missing %	Non-White Missing	Non-White Missing %
Race is White	28	2.0	0	0.0	0	0.0
Age	0	0.0	0	0.0	0	0.0
Gender	0	0.0	0	0.0	0	0.0
Country of Origin	9	0.6	7	0.6	1	0.5
Primary Language	2	0.1	1	0.1	1	0.5
Interpreter	5	0.4	4	0.4	1	0.5
Medical History	0	0.0	0	0.0	0	0.0
Stroke Type	0	0.0	0	0.0	0	0.0
Patient Arrival Mode	83	5.9	68	5.9	12	5.4
Systolic BP	64	4.6	55	4.8	8	3.6
Diastolic BP	72	5.1	57	4.9	14	6.3
Glucose Blood Level	103	7.3	83	7.2	19	8.5
Disposition	0	0.0	0	0.0	0	0.0
Mortality (30 days)	24	1.7	19	1.6	4	1.8
Length of Stay	0	0.0	0	0.0	0	0.0
Ambulatory state (prior)	1	0.1	1	0.1	0	0.0
Ambulatory state (discharge)	288	20.5	242	20.9	34	15.3
Readmission (30 days)	10	0.7	8	0.7	2	0.9
A1C	459	32.6	381	32.9	71	31.8
LDL	384	27.3	314	27.1	59	26.5
EF	341	24.2	280	24.2	54	24.2
Antihypertensives in ED	81	5.8	65	5.6	16	7.2
LKW to Arrival	337	23.9	272	23.5	58	26.0
Door to CT	310	22.0	262	22.6	44	19.7
Intubation in ED	68	4.8	51	4.4	17	7.6

Table S2: Frequency and percent of missing data for the acute ischemic stroke cohort

Variable	Whole Missing	Whole Missing %	White Missing	White Missing %	Non-White Missing	Non-White Missing %
Race is White	22	2.2	0	0.0	0	0.0
Age	0	0.0	0	0.0	0	0.0
Gender	0	0.0	0	0.0	0	0.0
Country of Origin	7	0.7	6	0.7	1	0.6
Primary Language	1	0.1	1	0.1	0	0.0
Interpreter	3	0.3	3	0.4	0	0.0
Medical History	0	0.0	0	0.0	0	0.0
Patient Arrival Mode	74	7.3	62	7.5	9	5.5
Systolic BP	19	1.9	14	1.7	4	2.4
Diastolic BP	56	5.5	45	5.4	10	6.1
Glucose Blood Level	74	7.3	61	7.3	12	7.3
Disposition	0	0.0	0	0.0	0	0.0
Mortality (30 days)	18	1.8	13	1.6	4	2.4
Length of Stay	0	0.0	0	0.0	0	0.0
Ambulatory state (prior)	1	0.1	1	0.1	0	0.0
Ambulatory state (discharge)	114	11.2	93	11.2	11	6.7
Readmission (30 days)	7	0.7	5	0.6	2	1.2
A1C	255	25.0	209	25.1	40	24.2
LDL	175	17.2	144	17.3	23	13.9
EF	175	17.2	141	17.0	29	17.6
Antihypertensives in ED	62	6.1	51	6.1	11	6.7
Initial NIHSS 1	260	25.5	207	24.9	46	27.9
NIHSS (24 hours)	311	30.5	255	30.7	48	29.1
LKW to Arrival	269	26.4	218	26.2	46	27.9
IV Thrombolytic received (this hospital)	1	0.1	1	0.1	0	0.0
Door to CT	226	22.2	189	22.7	34	20.6

Table S3: Frequency and percent of missing data for the transient ischemic attack cohort

Variable	Whole Missing	Whole Missing %	White Missing	White Missing %	Non-White Missing	Non-White Missing %
Race is White	1	0.5	0	0.0	0	0.0
Age	0	0.0	0	0.0	0	0.0
Gender	0	0.0	0	0.0	0	0.0
Country of Origin	0	0.0	0	0.0	0	0.0
Primary Language	0	0.0	0	0.0	0	0.0
Interpreter	1	0.5	1	0.5	0	0.0
Medical History	0	0.0	0	0.0	0	0.0
Patient Arrival Mode	3	1.4	2	1.0	1	5.3
Systolic BP	36	16.7	34	17.4	2	10.5
Diastolic BP	10	4.6	7	3.6	3	15.8
Glucose Blood Level	18	8.3	14	7.1	4	21.1
Disposition	0	0.0	0	0.0	0	0.0
Mortality (30 days)	3	1.4	3	1.5	0	0.0
Mortality (90 days)	6	2.8	6	3.1	0	0.0
Length of Stay	0	0.0	0	0.0	0	0.0
Ambulatory state (prior)	0	0.0	0	0.0	0	0.0
Ambulatory state (discharge)	100	46.3	94	48.0	5	26.3
Readmission (30 days)	2	0.9	2	1.0	0	0.0
A1C	92	42.6	86	43.9	6	31.6
LDL	80	37.0	74	37.8	6	31.6
EF	71	32.9	63	32.1	8	42.1
Antihypertensives in ED	12	5.6	8	4.1	4	21.1
Initial NIHSS 1	66	30.6	59	30.1	6	31.6
LKW to Arrival	20	9.3	18	9.2	2	10.5
EKG	8	3.7	5	2.6	3	15.8
TTE	8	3.7	5	2.6	3	15.8
TEE	29	13.4	23	11.7	6	31.6
CT Angio	9	4.2	6	3.1	3	15.8
MRA	73	33.8	65	33.2	7	36.8

Table S4: Frequency and percent of missing data for the intracerebral hemorrhage cohort

Variable	Whole Missing	Whole Missing %	White Missing	White Missing %	Non-White Missing	Non-White Missing %
Race is White	3	2.5	0	0.0	0	0.0
Age	0	0.0	0	0.0	0	0.0
Gender	0	0.0	0	0.0	0	0.0
Country of Origin	1	0.8	1	1.1	0	0.0
Primary Language	1	0.8	0	0.0	1	3.6
Interpreter	1	0.8	0	0.0	1	3.6
Medical History	0	0.0	0	0.0	0	0.0
Patient Arrival Mode	4	3.3	2	2.2	2	7.1
Systolic BP	5	4.1	4	4.4	1	3.6
Diastolic BP	5	4.1	5	5.5	0	0.0
Glucose Blood Level	7	5.7	6	6.6	1	3.6
Disposition	0	0.0	0	0.0	0	0.0
Mortality (30 days)	3	2.5	3	3.3	0	0.0
Length of Stay	0	0.0	0	0.0	0	0.0
Ambulatory state (prior)	0	0.0	0	0.0	0	0.0
Ambulatory state (discharge)	50	41.0	39	42.9	11	39.3
Readmission (30 days)	1	0.8	1	1.1	0	0.0
A1C	72	59.0	56	61.5	16	57.1
Antihypertensives in ED	6	4.9	6	6.6	0	0.0
LKW to Arrival	31	25.4	21	23.1	9	32.1
Door to CT	8	6.6	6	6.6	2	7.1
Glasgow Coma Scale	20	16.4	13	14.3	7	25.0
Intubation in ED	11	9.0	10	11.0	1	3.6
EVD Placement	7	5.7	7	7.7	0	0.0
Decompressive Craniectomy	7	5.7	7	7.7	0	0.0
Hematoma Evacuation	7	5.7	7	7.7	0	0.0

Table S5: Frequency and percent of missing data for the subarachnoid hemorrhage cohort

Variable	Whole Missing	Whole Missing %	White Missing	White Missing %	Non-White Missing	Non-White Missing %
Race is White	2	4.9	0	0.0	0	0.0
Age	0	0.0	0	0.0	0	0.0
Gender	0	0.0	0	0.0	0	0.0
Country of Origin	1	2.4	0	0.0	0	0.0
Primary Language	0	0.0	0	0.0	0	0.0
Interpreter	0	0.0	0	0.0	0	0.0
Medical History	0	0.0	0	0.0	0	0.0
Patient Arrival Mode	2	4.9	2	6.5	0	0.0
Systolic BP	3	7.3	2	6.5	1	12.5
Diastolic BP	1	2.4	0	0.0	1	12.5
Glucose Blood Level	3	7.3	1	3.2	2	25.0
Disposition	0	0.0	0	0.0	0	0.0
Mortality (30 days)	0	0.0	0	0.0	0	0.0
Length of Stay	0	0.0	0	0.0	0	0.0
Ambulatory state (prior)	0	0.0	0	0.0	0	0.0
Ambulatory state (discharge)	23	56.1	15	48.4	7	87.5
Readmission (30 days)	0	0.0	0	0.0	0	0.0
Antihypertensives in ED	1	2.4	0	0.0	1	12.5
LKW to Arrival	13	31.7	11	35.5	1	12.5
Door to CT	1	2.4	0	0.0	1	12.5
Glasgow Coma Scale	7	17.1	2	6.5	5	62.5
Intubation in ED	1	2.4	0	0.0	1	12.5
EVD Placement	1	2.4	0	0.0	1	12.5
Aneurysm Coiling	1	2.4	0	0.0	1	12.5
Aneurysm Clipping	2	4.9	1	3.2	1	12.5
CT Angio	1	2.4	0	0.0	1	12.5
MRA	16	39.0	10	32.3	4	50.0

Table S6: Reasons for not administering IV thrombolysis

	White (n=620)	Non-White (n=112)
Elevated blood pressure (SBP>185 mm Hg or DBP>110 mm Hg) despite treatment	11 (1.3%)	0
Recent intracranial or spinal surgery or significant head trauma or prior stroke in previous 3 months	24 (3.9%)	8 (7.1%)
History of previous intracranial hemorrhage, intracranial neoplasm, arteriovenous malformation or aneurysm	11 (1.8%)	3 (2.7%)
Active internal bleeding	2 (0.3%)	0
Acute bleeding diathesis (low platelet count, increased PTT, INR≥1.7, or use of NOAC)	76 (12.3%)	7 (6.3%)
Symptoms suggest subarachnoid hemorrhage	1 (0.2%)	0
CT demonstrates multilobar infarction (hypodensity>1/3 cerebral hemisphere)	0	1 (0.9%)
Care-team unable to determine eligibility	316 (51%)	75 (67%)
IV or IA thrombolysis/thrombectomy at an outside hospital prior to arrival	8 (1.3%)	2 (1.8)
Life expectancy<1 year or severe co-morbid illness or CMO on admission	10 (1.6%)	0
Pregnancy	0	0
Patient/family refusal	7 (1.1%)	2 (1.8%)
Stroke severity too mild (non-disabling)	144 (23.2%)	13 (11.6%)
Seizure at onset with postictal residual neurological impairments	0	1 (0.9%)
Major surgery or serious trauma within previous 14 days	7 (1.1%)	0
Recent gastrointestinal or urinary tract hemorrhage (within previous 21 days)	3 (0.5%)	0
