# Supplementary figures and images for: Racial Impact on Inpatient Stroke Quality of Care in Two Community Hospitals
Source: J Clin Med. 2023 Dec 13;12(24):7654. doi: 10.3390/jcm12247654 (PMC10743521; doi:10.3390/jcm12247654)

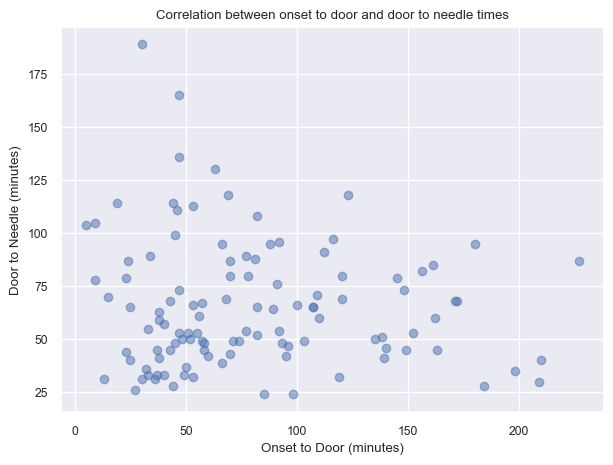

Supplement: Supplementary file 1 [file jcm-12-07654-s001.zip › Supplemental Figure S1.png]
